# Comparison of external R&D and internal R&D: Based on the perspective of S&T development of China’s pharmaceutical manufacturing industry

**DOI:** 10.1371/journal.pone.0270271

**Published:** 2022-06-22

**Authors:** Di Wu, Su Wang, Senhao Chang, Guiyu Lian, Yuwen Chen

**Affiliations:** 1 School of Business Administration, Shenyang Pharmaceutical University, Shenyang, Liaoning, China; 2 Shenyang Pharmaceutical and Large Health Industry Development Strategy Research Base, Shenyang, Liaoning, China; Institute for Advanced Sustainability Studies, GERMANY

## Abstract

**Purpose:**

The science and technology (S&T) innovation in China’s pharmaceutical industry has entered a bottleneck. The choice between external and internal research and development (R&D) has become a significant challenge for S&T development. To provide scientific suggestions for companies to choose an R&D strategy and enhance S&T development, we analyzed and compared the impacts of two R&D strategies on S&T output.

**Methods:**

We selected the data related to China’s pharmaceutical manufacturing industry from 2000 to 2019, established regression equations by the E-G two-step method, and used the VAR model for impulse responses and variance decompositions to research the relationship between two R&D strategies and S&T output.

**Results:**

There is a stable long-term equilibrium relationship between two R&D strategies, including external and internal R&D, and S&T output in China’s pharmaceutical manufacturing industry. When internal R&D increases by 1%, S&T output increases by 0.7382% with a 5-year lag. When S&T output increases by 1%, external R&D increases by 2.0749% with a 2-year lag.

**Conclusion:**

Compared with external R&D, internal R&D can boost S&T output.

## Introduction

On June 1, 2021, the Patent Law of the People’s Republic of China (2020 Revision) came into effect [1]. The new patent law has increased protection for pharmaceutical patents. It has added incentive systems such as the pharmaceutical patent term compensation system and pharmaceutical patent linkage system, which has led to the pharmaceutical industry attaching greater value to pharmaceutical patent protection [2]. Pharmaceutical knowledge output not only represents the knowledge reserve capacity of the pharmaceutical manufacturing industry but also reflects the science and technology (S&T) innovation power of the pharmaceutical industry [3]. As of now, the overall level of S&T innovation in China is in the predicament of "low quality and low efficiency", the utilization rate of research and development (R&D) resources is gradually decreasing, and the growth rate of the knowledge economy is slowing down [4]. The main reason is that the lack of capital and staffing prevents companies from implementing large-scale research projects independently [5]. From Schumpeter’s recombination perspective, most technological innovations are driven by recombining different technology elements [6]. Therefore, aiming to overcome these challenges, pharmaceutical companies are increasingly turning to external resources. They are gradually opening up their organizational boundaries and reorganizing their R&D systems [7]. We usually refer to this R&D strategy as external R&D.

In order to improve the degree of external R&D, companies have adjusted their internal R&D processes. However, with the continuous development of external R&D, companies are increasingly relying on external cooperation in the R&D process for S&T innovation. As a result, companies are gradually neglecting the importance of traditional internal R&D, which has led to a decline in their internal S&T innovation capabilities. In addition, external R&D companies face many problems, including complex cooperation management, insufficient knowledge absorption capacity, and leakage of technical knowledge. Therefore, a company’s reliance on external R&D can adversely affect its ability to innovate in S&T [8]. Claudio Cruz-Cázares purported that the main driver of S&T innovation is the intensity of internal R&D [9]. As a result, the impact of internal R&D on the innovation performance of companies has received much attention [10]. How companies choose between external and internal R&D has become a new challenge for the development of S&T innovation at present. We explore the impact of internal and external R&D on S&T output in the pharmaceutical manufacturing industry from the perspective of technology development. The contribution of this paper is to provide scientific suggestions for pharmaceutical companies’ managers to choose an R&D strategy and to promote the development of S&T innovation within the pharmaceutical manufacturing industry.

## External and internal R&D

### External R&D

External R&D refers to the spontaneous R&D cooperation between companies and universities, research institutes, and other companies according to their interests [11]. The two most common forms of external R&D are Contract Research Organizations (CROs) and Industry-University-Research Institutes (IURs). In technical terms, a CRO is described as an independent contractor for the sponsor who assumes one or more of the sponsor’s obligations [12]. CROs have distinctive characteristics, including specialization and efficiency [13]. The IURs refer to external R&D for which universities and research institutes are collaborators. Its distinctive feature is that each partner can contribute to R&D activities more comprehensively based on the strengths that they bring to the table [14]. Banerjee et al. found that to avoid competition, CROs intentionally choose their sites away from other CROs and thus occupy the partial market [15]. Therefore, there is a minimal possibility of cooperation between CROs. They cannot share knowledge among themselves, so they cannot impact the development of S&T together. In contrast, Zhou et al. concluded that the growth of the CRO industry in China has boosted the development of the Chinese pharmaceutical industry [16]. Thus it has increased the opportunities for international collaboration. Based on this, the government pays more attention to property protection, thus driving the development of S&T. Wu et al. developed a multi-equation time series model analyzing relevant panel data [17]. The results indicate that IUR can positively impact economic performance as well as S&T output, but the magnitude is low. Yuan et al. explored the relationship between cooperative R&D and technological upgrading by gathering data from Chinese manufacturing industries by subsectors [18]. The results highlight that cooperative R&D positively impacts technological upgrading. Jiang et al. used interprovincial panel data from 2009 to 2014 to explore the relationship between IUR intensity and S&T innovation capacity [19]. The results highlight an "inverted U-shaped" relationship between the two variables.

### Internal R&D

Internal R&D refers to the R&D activities carried out by the company itself without the help of external forces. It is characterized by the independence and autonomy to pursue S&T innovation [20]. Cai et al. analyzed balanced data from 341 electronics manufacturing companies [21]. The results conclude that internal R&D investment positively affects company performance, while external technology acquisition does not. Anzola-Román et al. explored the Average Marginal Effects (AMEs) by analyzing panel data for causal effects [22]. The study results confirm the positive effects of internal R&D and exogenous innovation behavior. Hara et al. purported that internal R&D has a positive impact on manufacturing technology upgrades [18]. Based on relevant panel data from 2000 to 2014, Chen et al. found that autonomous innovation can contribute to the advanced industrial structure in China [23]. Bao used the data from 24 large and medium-sized industrial companies in China’s manufacturing industry to examine the correlation between internal R&D and S&T innovation [24]. The results highlight that internal R&D is the main force driving S&T innovation in China’s manufacturing industry. Liu conducted an econometric test using relevant panel data and found that the effect of internal R&D on S&T output is not significant but has a positive impact on economic growth [25].

## A brief comment

China’s pharmaceutical manufacturing industry is experiencing a rapid development of external R&D, often represented by CROs and IURs. As a result, most pharmaceutical companies have gradually become dependent on an external R&D strategy, resulting in a gradual decline of internal R&D capability in the pharmaceutical manufacturing industry. Currently, China’s pharmaceutical S&T innovation is gradually entering a bottleneck period, and the choice between external and internal R&D strategies has become a significant challenge to the development of S&T innovation. The existing literature only analyzed the impact of one R&D strategy (external R&D or internal R&D) on S&T output, and few scholars have compared the impact of the two R&D strategies on S&T output. Furthermore, the available findings did not reach a consensus on the impact of the two R&D strategies on S&T output, and there was a lack of relevant studies for the pharmaceutical manufacturing industry. Therefore, it was critical and relevant to study the relationship between the two R&D strategies and S&T output in the pharmaceutical manufacturing industry. In this study, we used the data related to the pharmaceutical manufacturing industry in China from 2000 to 2019 to establish regression equations by the two-step E-G method. We used Vector Auto-Regressive (VAR) models for impulse responses and variance decompositions to compare and analyze the relationship between the two R&D strategies and S&T output.

## Methodology and data collection

### Methodology

We empirically compared external and internal R&D strategies in the Chinese pharmaceutical manufacturing industry using a VAR model. The model was proposed in 1980 by Sims, a renowned econometrician, and is commonly used for studying the dynamic prediction and description of a complex time series by disturbances in the random variables. It has been widely used in economics and other research fields [26]. In addition, the model is well suited for the study of interactions between more than two variables.

### Data collection

We empirically investigated the interrelationship between two R&D strategies and S&T output in the Chinese pharmaceutical manufacturing industry. The selected explanatory variables and explained variables are shown in Table 1.

**Table 1:** Variables information.

Variable	Metric (unit)	Index explanation	Symbol
External R&D explanatory variable	External expenditure of R&D expenditure (ten thousand yuan)	External R&D	COOP
Internal R&D explanatory variable	Internal expenditure of R&D funds (ten thousand yuan)	Internal R&D	INDE
Explained variable	Number of valid invention patents (pieces)	S&T output	PAT

Based on the available data, we selected relevant data from the “China Statistical Yearbook on High-Technology Industry” and the “China Statistical Yearbook on Science and Technology” from 2000 to 2019. In order to attenuate the multicollinearity and heteroskedasticity of the model, the data were logarithmically processed in this study. They were noted as LNCOOP, LNINDE, and LNPAT, respectively. Table 2 presents the descriptive statistics for each variable.

**Table 2:** Descriptive statistics.

Variable	N	Mean	S.D.	Min	Max
LNCOOP	60	5.4178	0.3554	4.8299	6.0438
LNINDE	60	6.0708	0.5667	5.1293	6.7850
LNPAT	60	3.7049	0.7717	2.4886	4.6804

Note: N represents the number of samples

## Empirical analysis

The empirical part of this study began by testing for the presence of unit roots in the series of variable pairs through the Augmented Dickey-Fuller (ADF) method. After determining the stationarity of the series, we conducted a cointegration test and a Granger causality test. Finally, we built a VAR model and used the impulse responses and variance decompositions to perform an in-depth analysis of each variable [27].

### Unit root test

Most of the economic time series data is not stationarity. In order to prevent the occurrence of the pseudo-regression phenomenon, we conducted the stationarity test on the corresponding data, and the test results are shown in Table 3. From Table 3, we can see that the original series of each variable are unsteady time series. After the first difference, the original hypothesis is rejected at the 5% significance level of all variables. In other words, all three variables are first-order single integer series and satisfy the conditions for conducting cointegration tests.

**Table 3:** LNCOOP, LNINDE, LNPAT, and their first difference ADF results.

Variable	Inspection type	ADF test statistic	t-Statistic at each significant level			Prob.	Test result
	(C,T,K)		1% level	5% level	10% level		
LNCOOP	(C,T,0)	-0.0038	-3.8573	-3.0403	-2.6605	0.9464	unstable
LNINDE	(C,T,0)	-1.4915	-3.8573	-3.0403	-2.6605	0.5148	unstable
LNPAT	(C,T,0)	-1.8108	-3.8573	-3.0403	-2.6605	0.3636	unstable
D(LNCOOP)	(C,0,0)	-7.2506	-3.8573	-3.0403	-2.6605	0.0000	stable
D(LNINDE)	(C,0,0)	-5.6526	-3.8573	-3.0403	-2.6605	0.0003	stable
D(LNPAT)	(C,0,0)	-7.7899	-4.5715	-3.6908	-3.2869	0.0000	stable

Note: 1) The test types (C,T,K) represent the intercept term, time trend, and lag order, respectively; 2) D() represents the first-order difference series of the corresponding indicators in parentheses.

### Cointegration test

In order to test the stationarity and equilibrium relationship among the variables, we chose the E-G two-step method for cointegration testing. We first established regression models among the variables by least squares with LNCOOP and LNINDE as explanatory variables and LNPAT as explained variables, respectively. The regression results are shown in Tables 4 and 5, and the regression equations are:

LnPAT=2.0749LnCOOP−7.5370 (1)

LnPAT=1.3546LnINDE−4.5188 (2)

**Table 4:** External R&D E-G two-step regression results.

Variable	Coefficient	Std. Error	t-Statistic	Prob.
LNCOOP	2.0749	0.1505	13.7856	0.0000
C	-7.5370	0.8171	-9.2235	0.0000
R-squared	0.9134	Mean dependent var		3.7049
Schwarz criterion	0.1205	Akaike info criterion		0.0209
F-statistic	190.0444	Hannan-Quinn criterion		0.0403
Prob. (F-statistic)	0.0000	Durbin-Watson stat		0.9020

**Table 5:** Internal R&D E-G two-step regression results.

Variable	Coefficient	Std. Error	t-Statistic	Prob.
LNINDE	1.3546	0.0327	41.3768	0.0000
C	-4.5188	0.1995	-22.6424	0.0000
R-squared	0.9895	Mean dependent var		3.7049
Schwarz criterion	-1.9976	Akaike info criterion		-2.0972
F-statistic	1712.0470	Hannan-Quinn criterion		-2.0777
Prob. (F-statistic)	0.0000	Durbin-Watson stat		2.2476

Secondly, we performed unit root tests on the residuals generated by Eqs (1) and (2) to test whether the relationship remains valid. Tables 6 and 7 illustrate the results. The p-values are less than 0.05, indicating that the residual terms of both regression equations reject the original hypothesis to a 5% confidence level. We can consider that the regression equation is smooth, and there is a cointegration relationship between LNCOOP and LNINDE and LNPAT, respectively. Thus, LNCOOP and LNINDE have a stable equilibrium relationship with LNPAT in the long run, respectively.

**Table 6:** ADF test results for external R&D residuals.

ADF test statistic	1% level	5% level	10% level	Prob.	Test result
-2.1551	-2.6923	-1.9601	-1.6070	0.0332	stable

**Table 7:** ADF test results for internal R&D residuals.

ADF test statistic	1% level	5% level	10% level	Prob.	Test result
-3.5122	-2.7282	-1.9662	-1.6050	0.0018	stable

According to Eqs (1) and (2), we proposed the following hypotheses.

1. When S&T output increases by 1%, external R&D increases by 2.0749%.
2. When external R&D increases by 1%, S&T output increases by 0.4819% (1/2.0749).
3. When S&T output increases by 1%, internal R&D increases by 1.3546%.
4. When internal R&D increases by 1%, S&T output increases by 0.7382% (1/1.3546).

### VAR lag period selection

There are various methods to choose the lag period. For example, sequential modified LR test statistic, Final Prediction Error Criterion (FPE), Akaike Information Criterion (AIC) [28], Schwarz Criterion (SC) [28, 29], Hannan-Quinn Criterion (HQ) [28, 30]. We modeled external and internal R&D on technology output in two separate VARs. Based on the experience of previous scholars, we selected the optimal lag order by AIC, SC, and HQ information criterions. Finally, we chose 2 and 5 as the lag order of both models, and the results are shown in Tables 8 and 9.

**Table 8:** Results of lag selection of external R&D and S&T output VAR models with different criterions.

Lags	Log L	LR	FPE	AIC	SC	HQ
0	-3.3876	NA	0.0062	0.5986	0.6975	0.6122
1	36.1521	66.8997	0.0001	-3.3503	-3.0534	-3.3093
2	43.7843	11.0242*	8.28e-05*	-3.7538*	-3.2591*	-3.6856*

**Table 9:** Results of lag selection of internal R&D and S&T output VAR models with different criterions.

Lags	Log L	LR	FPE	AIC	SC	HQ
0	24.2475	NA	0.0001	-2.9663	-2.8719	-2.9673
1	55.2202	49.5563	4.89e-06	-6.5626	-6.2794	-6.5657
2	64.6176	12.5299*	2.49e-06	-7.2823	-6.8103	-7.2873
3	71.9268	7.7964	1.77e-06	-7.7235	-7.0627	-7.7306
4	78.4472	5.2163	1.57e-06	-8.0596	-7.2099	-8.0686
5	95.8012	9.2554	4.11e-06*	-9.8401*	-8.8016*	-9.8512*

Note: 1) * indicates lag order selected by the criterion; 2) NA indicates that this column is not applicable

### Granger causality test

Further, we performed Granger causality tests on LNPAT for LNCOOP and LNINDE separately to explore if there is a long-term two-way causal relationship between the two R&D strategies. Table 10 shows that the variable LNPAT is the Granger cause of the variable LNCOOP, and the opposite is not valid. It indicates that S&T output is the Granger cause of external R&D, and hypothesis (1) holds. Table 11 shows that the variable LNINDE is the Granger cause of the variable LNPAT, and the opposite is invalid. Therefore, independent R&D is the Granger cause of S&T output, and hypothesis (4) holds.

**Table 10:** Results of granger causality test between LNCOOP and LNPAT.

Lags	Null hypothesis	Prob.	Critical result
2	LNPAT does not Granger Cause LNCOOP	0.0112	refuse
2	LNCOOP does not Granger Cause LNPAT	0.8248	accept

**Table 11:** Results of granger causality test between LNINDE and LNPAT.

Lags	Null hypothesis	Prob.	Critical result
5	LNPAT does not Granger Cause LNINDE	0.1608	accept
5	LNINDE does not Granger Cause LNPAT	0.0121	refuse

### VAR model test

**Fig 1:**
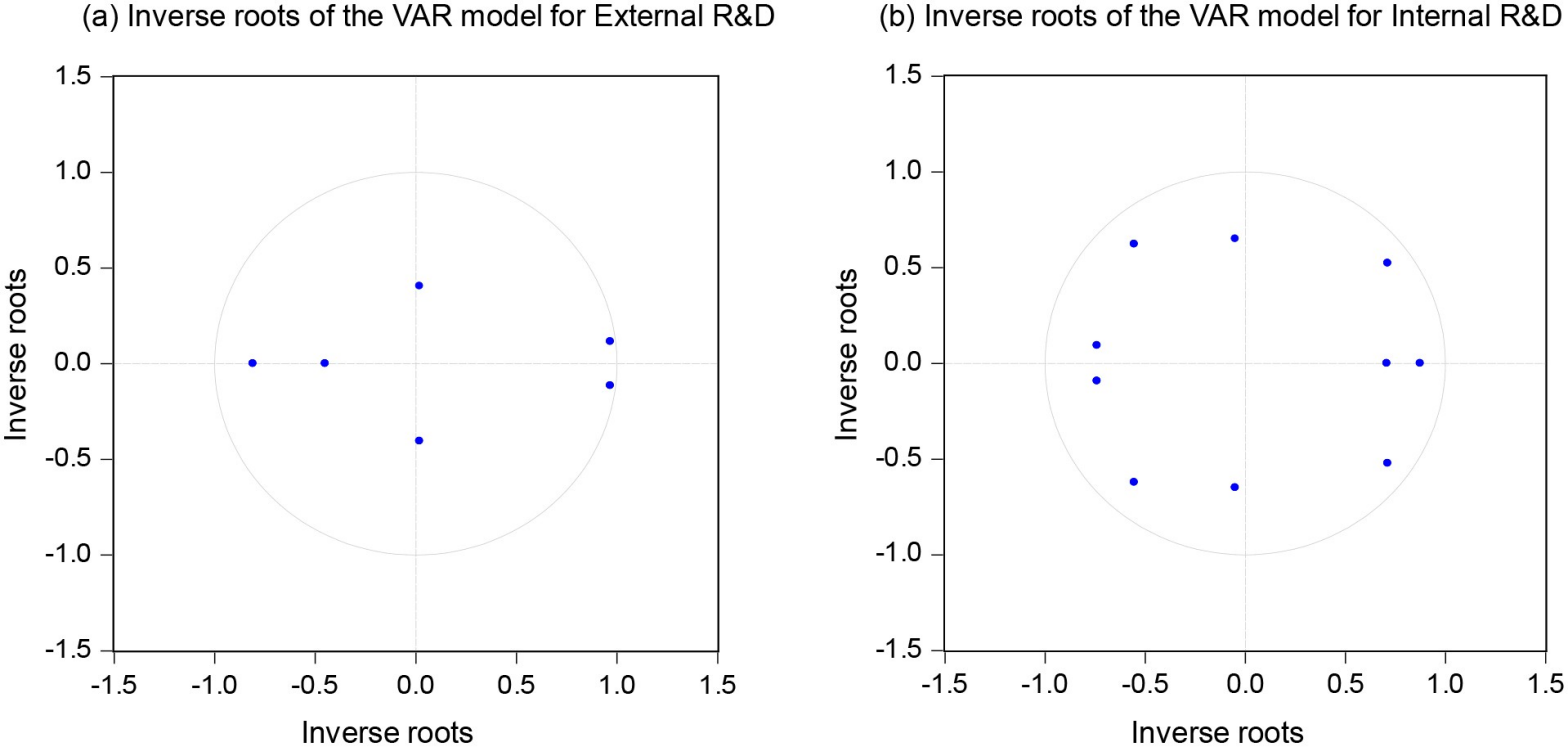
Stability tests for external and internal R&D VAR models.

Based on the results of the above lag order tests, we set the lag of the external R&D model to 2 and the lag of the internal R&D model to 5. The VAR models were constructed using Eviews 10.0, and the results are shown in Fig 1. All inverse roots are within the unit circle, indicating that the two models are stable and neither has correlation or heteroskedasticity.

### Impulse responses

**Fig 2:**
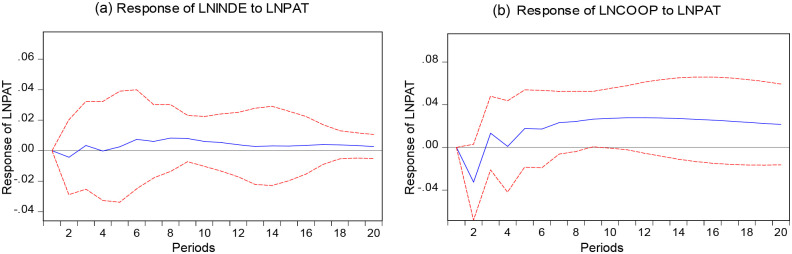
Impulse responses of LNINDE, LNCOOP to LNPAT.

We obtained the response trajectories of different exponential shocks between LNCOOP, LNINDE, and LNPAT by Eviews 10.0. The impulse responses’ figures of LNINDE, LNCOOP to LNPAT are shown in Fig 2. The trends of the effects of LNINDE and LNCOOP on LNPAT are similar in Fig 2. In the first 4 periods, the effect of LNINDE on LNPAT is first negative and then positive. In periods 5 and later, the effect of LNINDE then remains positive. Compared with LNINDE in Fig 2(a), the impact of LNCOOP on LNPAT fluctuates more in Fig 2(b).

**Fig 3:**
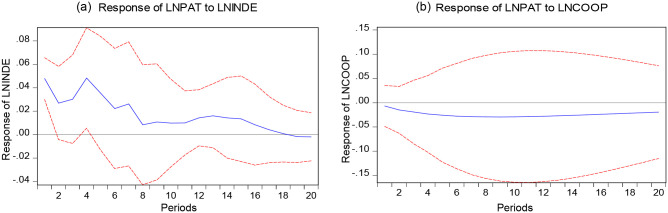
Impulse responses of LNPAT to LNINDE, LNCOOP. Note: The red dashed part is the confidence band with plus or minus two standard deviations, and the solid blue part is the calculated value.

The impulse responses of LNPAT to LNINDE and LNCOOP are shown in Fig 3. The impact effect of LNPAT on LNINDE shows an overall decreasing trend with an enormous change in Fig 3(a). The 18 period is the turning point of the impact effect from positive to negative. In Fig 3(b), the impact effect of LNPAT on LNCOOP is always negative, and the fluctuation of the effect is slight, almost in a straight line from period 6 onward. Due to this, we affirm that LNPAT is not sensitive to the impulse responses of LNCOOP.

### Variance decompositions

**Fig 4:**
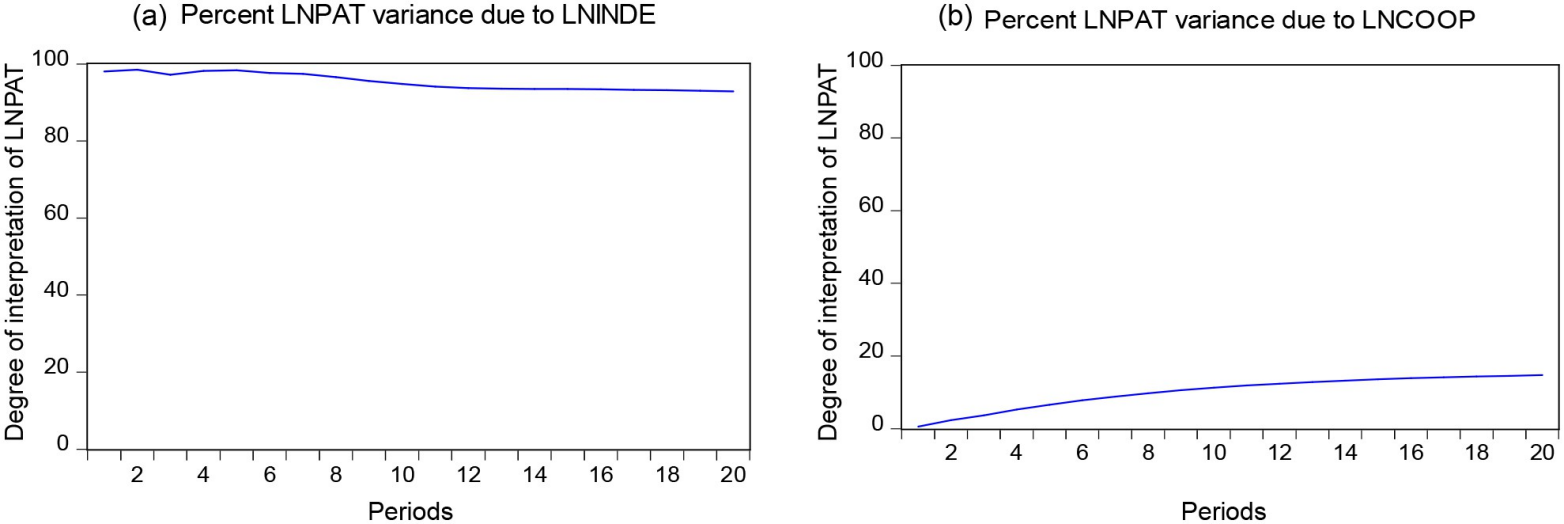
Variance decompositions results of LNINDE, LNCOOP on LNPAT.

Variance decompositions can analyze the degree of contribution of the changes in the explanatory variables to the shocks in the explained variables, determine the importance of the explanatory variables to the impact of the explained variables, and predict the future trends of the explanatory explained variables. Fig 4 shows the variance decompositions of LNINDE and LNCOOP on LNPAT. Comparing Fig 4(a) and 4(b), LNINDE explains LNPAT much more than LNCOOP. This finding is the same as the result of the Granger causality test. On this basis, LNPAT changes with the change of LNINDE.

**Fig 5:**
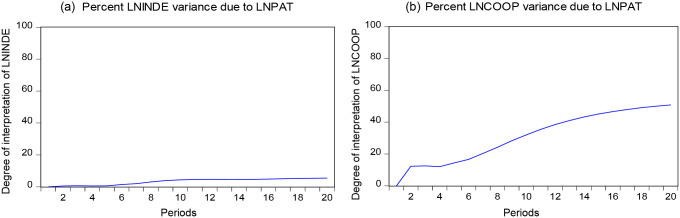
Variance decompositions results of LNPAT on LNINDE, LNCOOP.

Fig 5 shows the variance decompositions of LNPAT for LNINDE and LNCOOP. LNPAT explains only 5% of LNINDE at the highest degree in Fig 5(a). While in Fig 5(b), LNPAT explains up to 50% of LNCOOP. This finding is consistent with the results of the Granger causality test, according to which when LNPAT changes, LNCOOP also makes corresponding changes with its trend.

## Results and discussion

**Fig 6:**
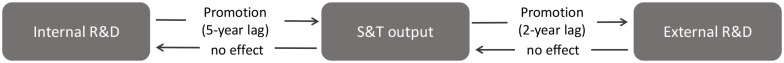
The relationship between two R&D Strategies and S&T output.

The results are as follows, and the relationship between the two R&D strategies and S&T output is shown in Fig 6.

1. Internal R&D has a positive contribution to S&T output, and there is a 5-year lag. When internal R&D increases by 1%, S&T output increases by 0.7382%. Conversely, there is no significant effect of S&T output on internal R&D. This may be due to the shortage of internal R&D funds due to long-term investment in S&T innovation with no return. If a company obtains a patent, it is often eager to file for patent subsidies or introduce the related product into the market to realize economic benefits for working capital, rather than immediately investing in the following internal R&D project.
2. S&T output positively promotes external R&D with a 2-year lag. When S&T output increases by 1%, external R&D increases by 2.0749%. Conversely, external R&D cannot effectively contribute to S&T output in pharmaceutical manufacturing. Perhaps this is due to external R&D involving more stakeholders, which results in the joint participation of multiple subjects in R&D will generally yield economic benefits rather than S&T innovation.
3. Comparatively to external R&D, only internal R&D can promote S&T output. It is possible because internal R&D can assist companies in capturing the market first and enhance competitiveness compared to external R&D. In contrast, external R&D tends to make companies dependent and lose the incentive to innovate. On this basis, the results of this study are in line with the current situation in the pharmaceutical manufacturing industry, such as over-reliance on external R&D leading to stagnation in S&T innovation.

## Conclusion

Aiming to examine the relationship between internal R&D, external R&D, and S&T output of the Chinese pharmaceutical manufacturing industry, we analyzed the data from 2000 to 2019 and drew the following conclusions. The internal R&D can effectively contribute to S&T output, but there is a 5-year lag. In contrast, an increase in S&T output expands internal R&D into collaborative R&D with a lag of only 2 years. We can see that S&T outputs require long-term and continuous support. When a patent is publicly licensed, the company that receives the patent license may collaborate with other pharmaceutical companies to obtain more excellent benefits.

As a result, we have provided two suggestions for pharmaceutical companies of various sizes.

1. Large companies should concentrate on improving internal R&D capabilities while conducting external R&D, establishing their R&D organizations, and increasing internal R&D expenditures. Companies should establish a proper "fit" with external R&D partners and continuously improve their internal R&D capabilities in the course of cooperation.
2. For Small and Medium Enterprises with weak internal R&D capability, they can first take advantage of CROs’ high professionalism and low cost to enhance their overall development through an external R&D strategy. Once the company has accumulated certain R&D funds and capabilities, it can focus its R&D efforts on its strengths to develop internal R&D capabilities. Improve the innovation capability over time to be on par with large companies.

## Limitations of this paper and future research recommendations

This study has the following two limitations. (1) This study uses an econometric model to investigate the correlation between two variables, likely to ignore the remaining important factors and lead to biased conclusions. (2) Due to limitations in data availability, the study uses the number of valid invention patents as an indicator of S&T output. Although inventions are one of the outputs of patents that best guarantee the quality of S&T, they do not adequately represent S&T.

Although this study concludes that external R&D cannot directly impact S&T output, the question remains whether external R&D can indirectly influence S&T output by influencing internal R&D or other indicators. Research on relevant topics can continue in future studies using mediating variables and other methods.

## Supporting information

S1 Dataset (DOC)

S1 Table (DOCX)
