# Supplementary material for: Comparison of external R&D and internal R&D: Based on the perspective of S&T development of China’s pharmaceutical manufacturing industry
Source: PLoS One. 2022 Jun 22;17(6):e0270271. doi: 10.1371/journal.pone.0270271 (PMC9216584; doi:10.1371/journal.pone.0270271)
Supplement: S1 Dataset — (DOC) [file pone.0270271.s001.doc]

**Table 1. Variables Information**

| **Variable** | **Metric (unit)** | **Index explanation** | **Symbol** |
| --- | --- | --- | --- |
| External R&D explanatory variable | External expenditure of R&D expenditure (ten thousand yuan) | External R&D | COOP |
| Internal R&D explanatory variable | Internal expenditure of R&D funds (ten thousand yuan) | Internal R&D | INDE |
| Explained variable | Number of valid invention patents (pieces) | S&T output | PAT |

Table 2. Descriptive Statistics

| **Variable** | **N** | **Mean** | **S.D.** | **Min** | **Max** |
| --- | --- | --- | --- | --- | --- |
| LNCOOP | 60 | 5.4178 | 0.3554 | 4.8299 | 6.0438 |
| LNINDE | 60 | 6.0708 | 0.5667 | 5.1293 | 6.7850 |
| LNPAT | 60 | 3.7049 | 0.7717 | 2.4886 | 4.6804 |

Note: N represents the number of samples

Table 3. LNCOOP, LNINDE, LNPAT, and Their First Difference ADF Results

| **Variable** | **Inspection type** | **ADF**  **test statistic** | **t-Statistic at each significant level** | | | **Prob.** | **Test result** |
| --- | --- | --- | --- | --- | --- | --- | --- |
|  | **(C,T,K)** |  | **1% level** | **5% level** | **10% level** |  |  |
| LNCOOP | (C,T,0) | -0.0038 | -3.8573 | -3.0403 | -2.6605 | 0.9464 | unstable |
| LNINDE | (C,T,0) | -1.4915 | -3.8573 | -3.0403 | -2.6605 | 0.5148 | unstable |
| LNPAT | (C,T,0) | -1.8108 | -3.8573 | -3.0403 | -2.6605 | 0.3636 | unstable |
| D(LNCOOP) | (C,0,0) | -7.2506 | -3.8573 | -3.0403 | -2.6605 | 0.0000 | stable |
| D(LNINDE) | (C,0,0) | -5.6526 | -3.8573 | -3.0403 | -2.6605 | 0.0003 | stable |
| D(LNPAT) | (C,0,0) | -7.7899 | -4.5715 | -3.6908 | -3.2869 | 0.0000 | stable |

Note: 1) The test types (C,T,K) represent the intercept term, time trend, and lag order, respectively; 2) D( ) represents the first-order difference series of the corresponding indicators in parentheses.

Table 4. External R&D E-G Two-step Regression Results

| **Variable** | **Coefficient** | **Std. Error** | **t-Statistic** | **Prob.** |
| --- | --- | --- | --- | --- |
| LNCOOP | 2.0749 | 0.1505 | 13.7856 | 0.0000 |
| C | -7.5370 | 0.8171 | -9.2235 | 0.0000 |
| R-squared | 0.9134 | Mean dependent var | | 3.7049 |
| Schwarz criterion | 0.1205 | Akaike info criterion | | 0.0209 |
| F-statistic | 190.0444 | Hannan-Quinn criterion | | 0.0403 |
| Prob. (F-statistic) | 0.0000 | Durbin-Watson stat | | 0.9020 |

Table 5. Internal R&D E-G Two-step Regression Results

| **Variable** | **Coefficient** | **Std. Error** | **t-Statistic** | **Prob.** |
| --- | --- | --- | --- | --- |
| LNINDE | 1.3546 | 0.0327 | 41.3768 | 0.0000 |
| C | -4.5188 | 0.1995 | -22.6424 | 0.0000 |
| R-squared | 0.9895 | Mean dependent var | | 3.7049 |
| Schwarz criterion | -1.9976 | Akaike info criterion | | -2.0972 |
| F-statistic | 1712.0470 | Hannan-Quinn criterion | | -2.0777 |
| Prob. (F-statistic) | 0.0000 | Durbin-Watson stat | | 2.2476 |

Table 6. ADF Test Results for External R&D Residuals

| **ADF test statistic** | **1% level** | **5% level** | **10% level** | **Prob.** | **Test result** |
| --- | --- | --- | --- | --- | --- |
| -2.1551 | -2.6923 | -1.9601 | -1.6070 | 0.0332 | stable |

Table 7. ADF Test Results for Internal R&D Residuals

| **ADF test statistic** | **1% level** | **5% level** | **10% level** | **Prob.** | **Test result** |
| --- | --- | --- | --- | --- | --- |
| -3.5122 | -2.7282 | -1.9662 | -1.6050 | 0.0018 | stable |

Table 8. Results of Lag Selection of External R&D and S&T Output VAR Models with Different Criterions

| **Lags** | **Log L** | **LR** | **FPE** | **AIC** | **SC** | **HQ** |
| --- | --- | --- | --- | --- | --- | --- |
| 0 | -3.3876 | NA | 0.0062 | 0.5986 | 0.6975 | 0.6122 |
| 1 | 36.1521 | 66.8997 | 0.0001 | -3.3503 | -3.0534 | -3.3093 |
| 2 | 43.7843 | 11.0242^*^ | 8.28e-05^*^ | -3.7538^*^ | -3.2591^*^ | -3.6856^*^ |

Table 9. Results of Lag Selection of Internal R&D and S&T Output VAR Models with Different Criterions

| **Lags** | **Log L** | **LR** | **FPE** | **AIC** | **SC** | **HQ** |
| --- | --- | --- | --- | --- | --- | --- |
| 0 | 24.2475 | NA | 0.0001 | -2.9663 | -2.8719 | -2.9673 |
| 1 | 55.2202 | 49.5563 | 4.89e-06 | -6.5626 | -6.2794 | -6.5657 |
| 2 | 64.6176 | 12.5299^*^ | 2.49e-06 | -7.2823 | -6.8103 | -7.2873 |
| 3 | 71.9268 | 7.7964 | 1.77e-06 | -7.7235 | -7.0627 | -7.7306 |
| 4 | 78.4472 | 5.2163 | 1.57e-06 | -8.0596 | -7.2099 | -8.0686 |
| 5 | 95.8012 | 9.2554 | 4.11e-06^*^ | -9.8401^*^ | -8.8016^*^ | -9.8512^*^ |

Note: 1) * indicates lag order selected by the criterion; 2) NA indicates that this column is not applicable

Table 10. Results of Granger Causality Test between LNCOOP and LNPAT

| **Lags** | **Null hypothesis** | **Prob.** | **Critical result** |
| --- | --- | --- | --- |
| 2 | LNPAT does not Granger Cause LNCOOP | 0.0112 | refuse |
| 2 | LNCOOP does not Granger Cause LNPAT | 0.8248 | accept |

Table 11.Results of Granger Causality Test between LNINDE and LNPAT

| **Lags** | **Null hypothesis** | **Prob.** | **Critical result** |
| --- | --- | --- | --- |
| 5 | LNPAT does not Granger Cause LNINDE | 0.1608 | accept |
| 5 | LNINDE does not Granger Cause LNPAT | 0.0121 | refuse |
