# Supplementary material for: Comparison of external R&D and internal R&D: Based on the perspective of S&T development of China’s pharmaceutical manufacturing industry
Source: PLoS One. 2022 Jun 22;17(6):e0270271. doi: 10.1371/journal.pone.0270271 (PMC9216584; doi:10.1371/journal.pone.0270271)
Supplement: S1 Table — (DOCX) [file pone.0270271.s002.docx]

**Original data table**

| **Years** | **Cooperative innovation** | **Independent innovation** | **Technology output** |
| --- | --- | --- | --- |
|  | **External expenditure of R&D expenditure(Ten thousand yuan)** | **Internal expenditure of R&D expenses(Ten thousand yuan)** | **Number of valid invention patents(Piece)** |
| 2000 | 67591 | 134669 | 414 |
| 2001 | 79583 | 192544 | 308 |
| 2002 | 113084 | 216359 | 484 |
| 2003 | 130091 | 276684 | 459 |
| 2004 | 142094 | 281812 | 902 |
| 2005 | 156537 | 399510 | 1134 |
| 2006 | 155178 | 525856 | 1965 |
| 2007 | 182210 | 658836 | 2482 |
| 2008 | 180419 | 790879 | 3170 |
| 2009 | 199598 | 1345385 | 6017 |
| 2010 | 177211 | 1226262 | 5672 |
| 2011 | 452929 | 2112462 | 10506 |
| 2012 | 338593 | 2833055 | 15058 |
| 2013 | 408187 | 3476553 | 19558 |
| 2014 | 482786 | 3903161 | 24799 |
| 2015 | 528596 | 4414567 | 31259 |
| 2016 | 600517 | 4884712 | 37463 |
| 2017 | 688622 | 5341769 | 41673 |
| 2018 | 979936 | 5808857 | 45766 |
| 2019 | 1106219 | 6095605 | 47910 |

**Corresponding variables are taken as logarithms**

| **Years** | **Cooperative innovation** | **Independent innovation** | **Technology output** |
| --- | --- | --- | --- |
|  | **LnCOOP** | **LnINDE** | **LnPAT** |
| 2000 | 4.8299 | 5.1293 | 2.6170 |
| 2001 | 4.9008 | 5.2845 | 2.4886 |
| 2002 | 5.0534 | 5.3352 | 2.6848 |
| 2003 | 5.1142 | 5.4420 | 2.6618 |
| 2004 | 5.1526 | 5.4500 | 2.9552 |
| 2005 | 5.1946 | 5.6015 | 3.0546 |
| 2006 | 5.1908 | 5.7209 | 3.2934 |
| 2007 | 5.2606 | 5.8188 | 3.3948 |
| 2008 | 5.2563 | 5.8981 | 3.5011 |
| 2009 | 5.3002 | 6.1288 | 3.7794 |
| 2010 | 5.2485 | 6.0886 | 3.7537 |
| 2011 | 5.6560 | 6.3248 | 4.0214 |
| 2012 | 5.5297 | 6.4523 | 4.1778 |
| 2013 | 5.6109 | 6.5411 | 4.2913 |
| 2014 | 5.6838 | 6.5914 | 4.3944 |
| 2015 | 5.7231 | 6.6449 | 4.4950 |
| 2016 | 5.7785 | 6.6888 | 4.5736 |
| 2017 | 5.8380 | 6.7277 | 4.6199 |
| 2018 | 5.9912 | 6.7641 | 4.6605 |
| 2019 | 6.0438 | 6.7850 | 4.6804 |
